# Prognostic significance of MUC2, CDX2 and SOX2 in stage II colorectal cancer patients

**DOI:** 10.1186/s12885-021-08070-6

**Published:** 2021-04-06

**Authors:** Sara Ribeirinho-Soares, Diana Pádua, Ana Luísa Amaral, Elvia Valentini, Daniela Azevedo, Cristiana Marques, Rita Barros, Filipa Macedo, Patrícia Mesquita, Raquel Almeida

**Affiliations:** 1 i3S - Instituto de Investigação e Inovação em Saúde, Universidade do Porto, Rua Alfredo Allen, 208, 4200-135 Porto, Portugal; 2 IPATIMUP - Institute of Molecular Pathology and Immunology, University of Porto, Porto, Portugal; 3 Centro Hospitalar de São João, Porto, Portugal; 4 Faculty of Medicine, University of Porto, Porto, Portugal; 5 IPO-C - Instituto Português de Oncologia de Coimbra Francisco Gentil, E. P. E, Coimbra, Portugal; 6 Biology Department, Faculty of Sciences of the University of Porto, Porto, Portugal

**Keywords:** Stage II colorectal cancer, Biomarkers, MUC2, CDX2, SOX2

## Abstract

**Background:**

Colorectal cancer (CRC) remains a serious health concern worldwide. Despite advances in diagnosis and treatment, about 15 to 30% of stage II CRC patients subjected to tumor resection with curative intent, develop disease relapse. Moreover, the therapeutic strategy adopted after surgery is not consensual for these patients. This supports the imperative need to find new prognostic and predictive biomarkers for stage II CRC.

**Methods:**

For this purpose, we used a one-hospital series of 227 stage II CRC patient samples to assess the biomarker potential of the immunohistochemical expression of MUC2 mucin and CDX2 and SOX2 transcription factors. The Kaplan-Meier method was used to generate disease-free survival curves that were compared using the log-rank test, in order to determine prognosis of cases with different expression of these proteins, different mismatch repair (MMR) status and administration or not of adjuvant chemotherapy.

**Results:**

In this stage II CRC series, none of the studied biomarkers showed prognostic value for patient outcome. However low expression of MUC2, in cases with high expression of CDX2, absence of SOX2 or MMR-proficiency, conferred a significantly worst prognosis. Moreover, cases with low expression of MUC2 showed a significantly clear benefit from treatment with adjuvant chemotherapy.

**Conclusion:**

In conclusion, we observe that patients with stage II CRC with low expression of MUC2 in the tumor respond better when treated with adjuvant chemotherapy. This observation supports that MUC2 is involved in resistance to fluorouracil-based adjuvant chemotherapy and might be a promising future predictive biomarker in stage II CRC patients.

**Supplementary Information:**

The online version contains supplementary material available at 10.1186/s12885-021-08070-6.

## Background

Colorectal cancer (CRC) is a serious health concern, being the third most commonly diagnosed cancer and the second most important cause of cancer-related deaths worldwide [1, 2]. Despite advances in diagnosis and treatment, about 15 to 30% of patients with stage II disease suffer from recurrent loco-regional disease or distant metastases within 5 years and their overall survival (OS) is around 70% [3, 4].

For non-metastasized CRC, which includes stage II patients, resective surgery is the main curative treatment. In some cases of rectal carcinoma, neo-adjuvant treatments are also administered [5]. After surgery, fluorouracil (5-FU)-based chemotherapy has been used to decrease the risk of relapse and increase survival of patients with resected CRC [6]. However, despite that disease-free survival (DFS) among patients with stage III CRC increases significantly with adjuvant chemotherapy regimens, the same is not observed in earlier stages of CRC [7]. Presently, the decision of giving adjuvant treatment based on the administration of fluoropyrimidine to stage II CRC patients is recommended to high risk patients with one or more risk factors: primary tumors diagnosed in T4; poorly differentiated grade, except if associated with mismatch repair (MMR) deficiency; presence of lymphovascular and/or perineural invasion; perforation and/or obstruction; close, undetermined or positive resection margins or less than 12 lymph nodes in the surgical resection specimen [8]. Patients with very high risk - microsatellite stable (MSS) and T4 or more than one corroborated risk factor - may be considered for the addition of oxaliplatin to fluoropyrimidine, whereas for patients with low-risk only follow-up is recommended (Labianca et al., 2013 and respective ESMO Guidelines Committee eUpdate, 2019) [9].

There is an urgent need of validated biomarkers that allow distinguishing the patients that will benefit from adjuvant chemotherapy thus supporting clinical decisions in stage II CRC. In the last years, there has been an intense investigation for new biomarkers, but unfortunately no optimal biomarker has been recognized in the clinic to find patients with a higher risk and predict relapse of stage II colon cancer.

Transcription factors (TFs) that control differentiation programs have been shown to be dysregulated in human cancers [10] and their altered expression patterns are many times significantly associated with patient’s prognosis [11] and drug sensitivity [12].

The caudal type homeobox 2 transcription factor (CDX2) is a master regulator of intestine-specific genes crucial for the balance between intestinal cell differentiation and proliferation [13, 14] and with a fundamental role in the maintenance of intestinal homeostasis [15]. CDX2 is highly expressed in normal colorectal epithelium, but its levels decrease in a subset of CRCs [15]. It was identified as prognostic and predictive biomarker for the response to chemotherapy in stages II and III CRC [16]. Additionally, in stage IV CRC, the absence of CDX2 expression anticipated poor patient survival [17, 18].

The oligomeric mucus/gel-forming mucin 2 (MUC2) is a molecular target of CDX2 [19] and has been demonstrated as a prognostic biomarker in CRC, namely in stage II cases [20–24]. Mucins are high-molecular weight epithelial glycoproteins with a high content of oligosaccharides O-glycosidically linked to tandem repeat peptides rich in threonine, serine, and proline [25]. There are two structurally and functionally distinct classes of mucins: secreted gel-forming mucins (MUC2, MUC5AC, MUC5B and MUC6) and transmembrane mucins (MUC1, MUC3A, MUC3B, MUC4, MUC12 and MUC17). The secreted gel-forming mucin, MUC2, is abundantly expressed in the intestinal mucosa, by goblet cells, and it is generally decreased in colorectal adenocarcinoma, except in mucinous carcinomas [26–29].

Sex-determining region Y-box transcription factor 2 (SOX2) is a member of the large SOX gene family, that includes transcription factors with known central roles in the regulation of developmental processes and cell type specification in the normal colorectal epithelium [30, 31]. In CRC, SOX2 de novo expression has been related to poorly differentiated and more invasive tumors and poor OS, particularly in cases having *BRAF*^*V600E*^ mutation [31]. Nevertheless, this prognostic significance is stage-dependent and it is only perceived in a subset of patients who did not receive adjuvant chemotherapy [32].

A common feature of biomarker identification is the lack of consistent results, particularly in early stage CRC. In this study, we wanted to assess the single and combined clinical value of CDX2, MUC2 and SOX2 in stage II CRC, in order to clarify the inconsistent results described in the literature.

## Methods

### Patients

This retrospective study initially included 322 consecutive cases diagnosed with stage II colorectal adenocarcinoma and subjected to surgery with curative intent in Centro Hospitalar S. João (CHSJ), Porto, Portugal, between January 2002 and December 2010. The final series of 227 patients excludes patients that: 1) were lost to follow-up; 2) were incorrectly staged; 3) died of post-operative complications; 4) had more than one type of cancer or 5) had a relapse during the first 6 months after surgery. Patients without or with insufficient tumor tissue available and those for whom clinical information could not be collected were also excluded. The clinicopathological features, treatment (described in Table 1) and follow-up data were obtained from both the patient institutional records at CHSJ and the hospital-based cancer registry. Patients were observed each 3 months in the 2 years after surgery and each 6 months or annually in the following years. All patients were followed up throughout the study period. Data were collected prospectively, between 2002 and 2016 (last follow-up), and analysed retrospectively. It is further indicated the administration of neo-adjuvant and adjuvant chemotherapy, presence of *BRAF*^*V600E*^ mutation and MMR status. The tumor tissue was earlier processed in the diagnostic routine following surgery.

**Table 1:** Clinicopathological characteristics of the 227 patients with stage II colorectal carcinoma included in this study

	***Frequency (n)***	***Percentage (%)***
**Patients**	227	
**Age (years)**		
Media	68.3 ± 11.5	
Range	23–92	
**Sex**		
Female	92	40.5
Male	135	59.5
**Histopathological grade**		
G1	4	1.8
G2	210	93.7
G3	10	4.5
Mucinous	3	
**Tumor Location**		
Proximal colon	75	33.5
Distal colon	107	47.8
Rectum	42	18.7
ND	3	
**Chemotherapy**		
Neo-Adjuvant		
Yes	11	4.8
No	216	95.2
Adjuvant		
Yes	34	15.4
No	187	84.6
ND	6	
**Resection margins**		
R0	179	98.9
R1/R2	2	1.1
ND	46	
***BRAF*** ^***V600E***^		
WT	186	86.1
MUT	30	13.9
ND	11	
**Mismatch repair status**		
MMR-deficient	91	43.5
MMR-proficient	118	56.5
ND	18	

### Immunohistochemical analyses of protein expression

Histological tissue was collected from surgical specimens using a standard protocol and fixed in buffered formalin. Two mm diameter cores from all tumors were transferred to tissue microarrays (TMAs) and further analysed. Representative areas of tumor tissue were selected from central areas of the tumor, avoiding necrotic or fibrotic foci, based on visual assessment of the hematoxylin-eosin-stained slides.

Immunohistochemistry (IHC) for MUC2 mucin, CDX2 and SOX2 transcription factors was performed following standard methodologies and described in Camilo et al., 2014 [33]. Briefly, after deparaffination in xylene for 10 min and rehydration, heat-induced epitope retrieval was carried out in an IHC-Tek Epitope Retrieval Steamer Set for 40 min with 10 mM citrate buffer, pH 6.0 (CDX2) or 10 mM EDTA, pH 8.0 (SOX2 and MUC2). Incubation with primary antibodies for MUC2 (1:50 dilution, CCP58 clone, DAKO, Glostrup, Denmark), CDX2 (1:50 dilution, CDX2–88 clone, Biogenex, California, USA) and SOX2 (1:50 dilution, SP76 clone, Cell Marque, California, USA) was performed overnight, at 4 °C. Detection was performed using the Dako REAL™ Envision™ Detection System Peroxidase/DAB+ (DAKO, Glostrup, Denmark) according to the manufacturer’s instructions and tissue sections were counterstained with Gill’s haematoxylin (Leica Microsystems, Bucks, UK), dehydrated, clarified and mounted. Normal colonic mucosa was used as a positive control for the expression of MUC2 and CDX2 and normal gastric mucosa for SOX2 expression. The IHC for CDX2 was evaluated regarding nuclear expression and cases where the tumor completely lacked or exhibited < 25% of CDX2-positive cancer cells, were considered CDX2-low [16]. MUC2 was evaluated regarding cytoplasmatic expression and the same expression criteria used for CDX2 were considered [24]. SOX2 was evaluated as nuclear expression and since SOX2 is not expressed in the normal colorectal mucosa, any expression in cancer cells above 5% was considered positive [33]. Images were acquired with a 20x amplification, using the light microscope Olympus with DP 25 camera and Cell B acquisition software, after performing white balance.

The IHC for the MMR proteins was carried out using antibodies for MLH1 (1:50 dilution, G168–728 clone, BD Pharmingen, New Jersey, USA), MSH2 (Pre-diluted, 25D12 clone, Leica Biosystems, Wetzlar, Germany), MSH6 (1:500, PU29 clone, Leica Biosystems, Wetzlar, Germany) and PMS2 (1:100, MOR4G clone, Leica Biosystems, Wetzlar, Germany), and the Leica Polymer Refine Detection kit on a Leica Bond-III Automated IHC stainer (Leica Microsystems, Wetzlar, Germany). The antigen retrieval for these four proteins was performed in BOND Epitope Retrieval Solution 2 (Leica Biosystems, Wetzlar, Germany) for 20 min. Normal colonic mucosa was used as a positive control for the expression of the MMR proteins. A tumor was considered positive for the expression of MMR proteins if at least one cancer cell showed nuclear staining, as previously reported by Koopman et al.*,* 2009 [34].

### DNA extraction from paraffin-embedded tissues

Haematoxylin and eosin (HE) staining was undertaken to guarantee that the tumor specimens tested contained more than 20% cancer cells, and areas enriched in malignant cells were identified before DNA extraction. Two slides with 10 μm were deparaffinized, dehydrated and macrodissected with a surgical blade from the two tissue sections. Genomic DNA was extracted with Cell Lysis solution (Citomed, Lisbon, Portugal) and digested with proteinase K 20 mg/mL (Thermo Fisher Scientific, Massachusetts, USA). Proteins were then precipitated with Protein Precipitation solution (Citomed, Lisbon, Portugal) and discarded. Isopropanol and glycogen were added to the genomic fraction in order to precipitate DNA. After a centrifugation step, the supernatant was carefully discarded and the pellet washed with ethanol. Pellets were rehydrated with autoclaved deionized water and stored at − 20 °C until use. The concentration of the extracted DNA was assessed using a Nano-Drop 1000 instrument (Thermo Fisher Scientific, Massachusetts, USA).

### Characterization of the *BRAF*^*V600E*^ mutation

Primary tumors were assessed for the presence of the *BRAF*^*V600E*^ mutation in genomic DNA extracted from the paraffin-embedded tissues. DNA was amplified with the Taq PCR Master Mix Kit (Qiagen, Hilden, Germany) using the forward: 5′-GGAAAGCATCTCACCTCATCC-3′, and the reverse: 5′-AACTCAGCAGCATCTCAGGGC-3′ primers (Sigma-Aldrich, Missouri, USA), designed for the exon 15 of the *BRAF* gene. Sterilized water was included as template negative control. PCR amplification was performed as following: an initial activation step at 95 °C for 15 min, three denaturation cycles at 95 °C for 30 s, a first 8-cycle stage, including denaturation at 95 °C for 30 s, annealing with touchdown temperature of 65 °C to 57 °C for 90 s and extension at 72 °C for 1 min, then an additional 32-cycle stage, including denaturation at 95 °C for 30 s, annealing at 60 °C for 30 s and extension at 72 °C for 1 min, and a final step of extension for 10 min at 72 °C. PCR products were analysed in a 2% agarose gel and stained with GelRed (Intron Biotechnology, South Korea) in order to confirm the presence of the expected 200 bp fragment.

PCR products were purified using the ExoSAP-IT Express PCR Product Cleanup reagent (Applied Biosystems, California, USA) and sequencing reactions were run using the BigDye Terminator v3.1 cycle sequencing Kit (Applied Biosystems, California, EUA) according to manufacturer’s instructions. Sequencing reaction products, using both forward and reverse primers, were purified with Sephadex (GE Healthcare, Illinois, EUA) and mixed with formamide. Sanger sequencing of all PCR products was subsequently conducted on an Applied Biosystems 3500 Genetic Analyzer (Thermo Fisher Scientific, Massachusetts, USA) and sequences were analysed with Applied Biosystems Quality Check software (Thermo Fisher Cloud). Tumors with the *BRAF*^*V600E*^ mutation were classified as mutant *BRAF* (versus wild type).

### Statistical analysis

This study followed the REMARK guidelines to report biomarkers (Table S1) [35]. Our objective was to study the association between the expression status of CDX2, MUC2 and SOX2 and the clinicopathological features of the patients (Table 2), for which we used different statistical tests. The t student test was used when comparing with age. Fisher’s exact test (2-sided) was used when comparing with sex, MMR status and BRAF^V600E^ mutation and chi-square (χ^2^) test was used when comparing with the histopathological grade and tumor location. Another objective was to assess the association between the expression status of CDX2, MUC2 and SOX2 and the risk of relapse. This was performed using the Kaplan-Meier method in order to generate DFS plots and the survival curves were compared using the log-rank test. DFS was defined as the time from surgery to the first event of either loco-regional recurrence or metastasis, or death from the same cancer. In order to evaluate if CDX2, SOX2 or MUC2 expression could predict response to adjuvant chemotherapy, DFS plots were generated according to the expression status of these proteins and administration or not of adjuvant chemotherapy. Cox proportional hazards model was used to calculate univariable hazard ratios (HR) and confidence intervals (CI) for disease recurrence. Differences were considered statistically significant when *P* value < 0.05. Statistical analysis was performed in IBM SPSS Statistics version 24.

**Table 2:** Clinicopathological data association with CDX2, MUC2 and SOX2 status in 227 patients with stage II colorectal carcinoma

	CDX2			MUC2			SOX2		
	Low	High	***P***	Low	High	***P***	–	+	***P***
Patients	33	194		164	63		184	43	
**Age (years)**									
Media	67.7 ± 11.8	68.4 ± 11.4	0.81	68.3 ± 11.7	68.2 ± 11.1	0.96	67.9 ± 11.5	69.9 ± 11.1	0.74
Range	35–87	23–92		23–92	37–83		23–92	37–87	
**Sex**									
Female	19	73	0.04*	65	27	0.65	72	20	0.39
Male	14	121		99	36		112	23	
**Histopathological grade (*n*** **= 224; ND = 3)**									
G1	0	4	< 0.001*	1	3	0.05	3	1	0.20
G2	25	185		157	53		173	37	
G3	7	3		6	4		6	4	
**Tumor Location (*n*** **= 224; ND = 3)**									
Proximal colon	16	59	0.09	48	27	0.13	58	17	0.22
Distal colon	14	93		83	24		92	15	
Rectum	3	39		30	12		32	10	
**Mismatch repair status (*n*** **= 209; ND = 18)**									
MMR-deficient	22	69	< 0.001*	68	23	0.75	77	14	0.37
MMR-proficient	7	111		85	33		93	25	
***BRAF***^**V600E**^ **(*n*** **= 216; ND = 11)**									
WT	25	161	0.17	132	54	1.00	152	34	0.80
MUT	7	23		21	9		24	6	
**CDX2**									
Low				23	10	0.83	26	7	0.81
High				141	53		158	36	
**MUC2**									
Low							137	27	0.13
High							47	16	

*Notes.* It is indicated the number of cases with low and high expression of CDX2 and MUC2 and negative (−) or positive (+) for SOX2, for all the clinicopathological categories, except age. *P* values (statistical significance threshold< 0.05) were obtained using Student’s t test for the continuous variable, Fisher’s exact test (2-sided) and Qui-square (χ^2^) test for categorical variables

* indicates comparisons with *P* < 0.05

## Results

### Clinicopathological features and expression of MUC2, CDX2 and SOX2 in a stage II CRC series

The clinicopathological features of this cohort of stage II CRC patients were described in Table 1.

The median age was 68.3 years (range, 23–92), 135 patients (59.5%) were men and 92 (40.5%) were women. Seventy-five (33.5%) tumors were located in the right colon (proximally), 107 (47.8%) were found distally (left colon) and 42 (18.7%) were in the rectum. Regarding treatment, 11 (4.8%) patients (all with rectal cancer) received neo-adjuvant chemotherapy and 34 (15.4%) patients received adjuvant chemotherapy. *BRAF*^*V600E*^ mutation status was assessed in 216 patients, 30 (13.9%) of each were mutated. For the rest of the tumors the material was insufficient for DNA extraction. MMR deficiency was evaluated by analysing the expression of the MMR proteins - MLH1, MSH2, MSH6 and PMS2 – by IHC. We were able to infer the MMR status of 209 patients. MLH1 expression was observed in 126 (60.3%) tumors and 83 (39.7%) showed loss of expression of this protein. Regarding MSH2 expression, 194 (92.8%) patients were positive and 15 (7.2%) were negative. MSH6 expression was found in 165 (78.9%) tumors and was absent in the other 44 (21.1%). Finally, concerning PMS2 expression, 170 (81.3%) tumors were positive whereas 39 (18.7%) were negative. In summary, 118 (56.5%) patients were MMR-proficient, based on the fact that they have not lost the expression of any of the four proteins, and 91 (43.5%) patients were MMR-deficient since they have lost the expression of at least one protein [36, 37].

**Fig. 1:**
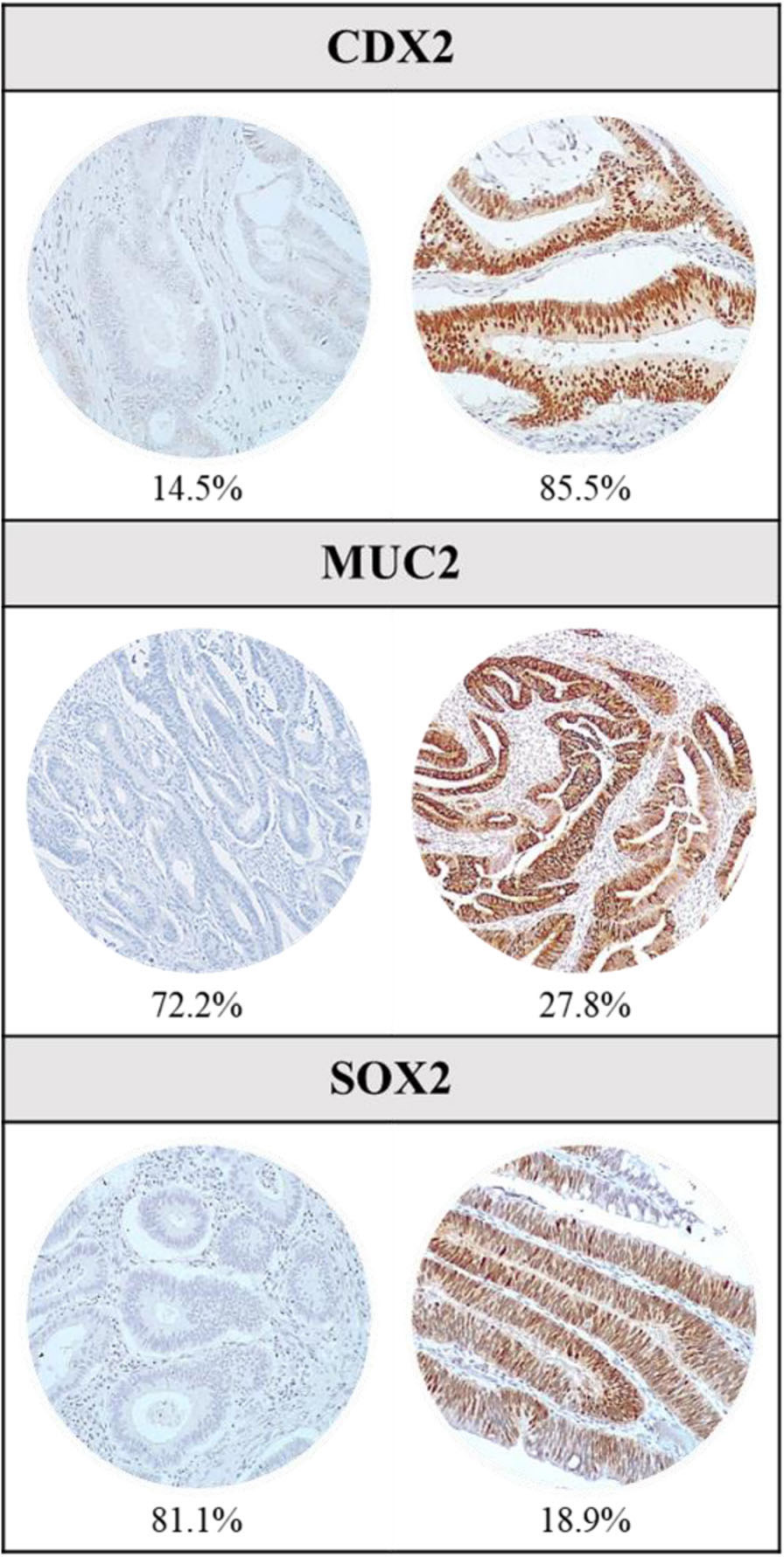
Representative immunostaining patterns for CDX2, MUC2 and SOX2 protein expression and respective percentages in the 227 stage II colorectal carcinomas evaluated

Analysis of CDX2, MUC2 and SOX2 expression was performed by IHC, in all 227 tumors (Fig. 1). CDX2 loss of expression was observed in 33 (14.5%) patients whereas MUC2 was expressed at low levels in 164 (72.2%) patients and at high levels in 63 (27.8%) patients (Table 2). SOX2 de novo expression was observed in 43 (18.9%) patients (Table 2).

In this series, CDX2 loss of expression was more common in females (*P* = 0.04) and it was strongly correlated with poorly differentiated tumors (*P* < 0.001) and with MMR-deficiency (*P* < 0.001). MUC2 and SOX2 expression did not show a significant correlation with any of the clinicopathological variables evaluated (Table 2), except for a borderline significant association between low expression of MUC2 and moderately/poorly differentiated tumors. The expression of the three biomarkers did not correlate between them.

### Prognostic significance of CDX2, MUC2 and SOX2 expression

In our series of 227 patients diagnosed with stage II CRC, the 5-year DFS was 81.0% and adjuvant chemotherapy was not associated with a significantly longer patient DFS (data not shown).

**Fig. 2:**
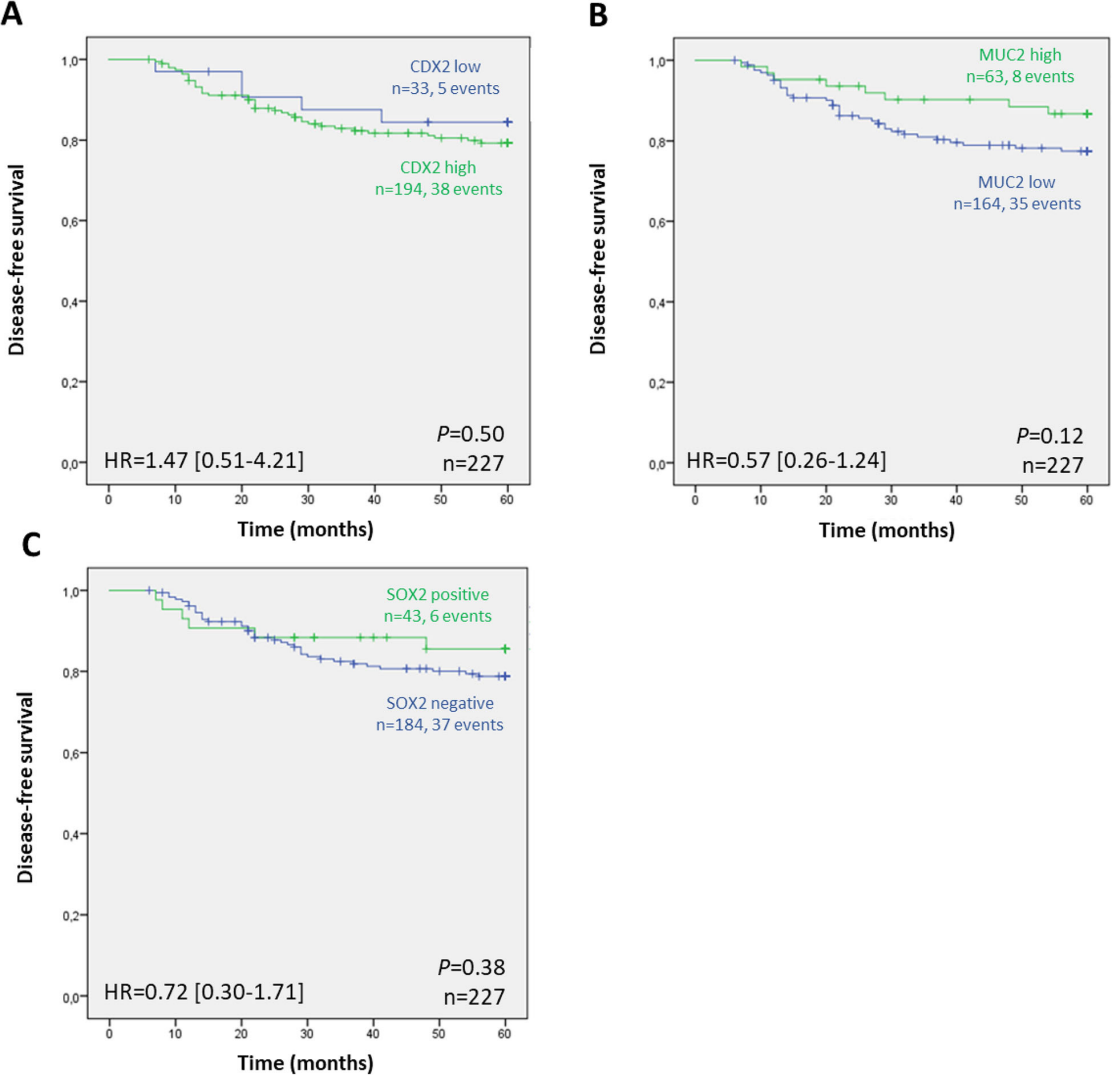
Kaplan-Meier curves showing the probability of disease-free survival in our series of patients with stage II colorectal cancer, according to **a** CDX2, **b** MUC2 and **c** SOX2 expression. The log-rank test was used to test for differences in survival between different levels of expression, while univariable Cox regression (Wald) was used to generate hazard ratios (HR) and 95% confidence intervals (CI), after adjustment for sex and tumor grade

We have calculated the univariable hazard ratios, using the Cox model, for the relevant clinicopathological features and molecular parameters described in Table 1, which did not give statistically significant results (Table S2). For this reason, multivariable analysis was not performed. Then we evaluated DFS according to the expression of CDX2, MUC2 and SOX2, using the Kaplan-Meyer method. Neither protein showed significant relevance as prognostic biomarker for patient DFS (Fig. 2), although for MUC2 there was a clear tendency for better DFS when tumors had high MUC2 expression.

**Fig. 3:**
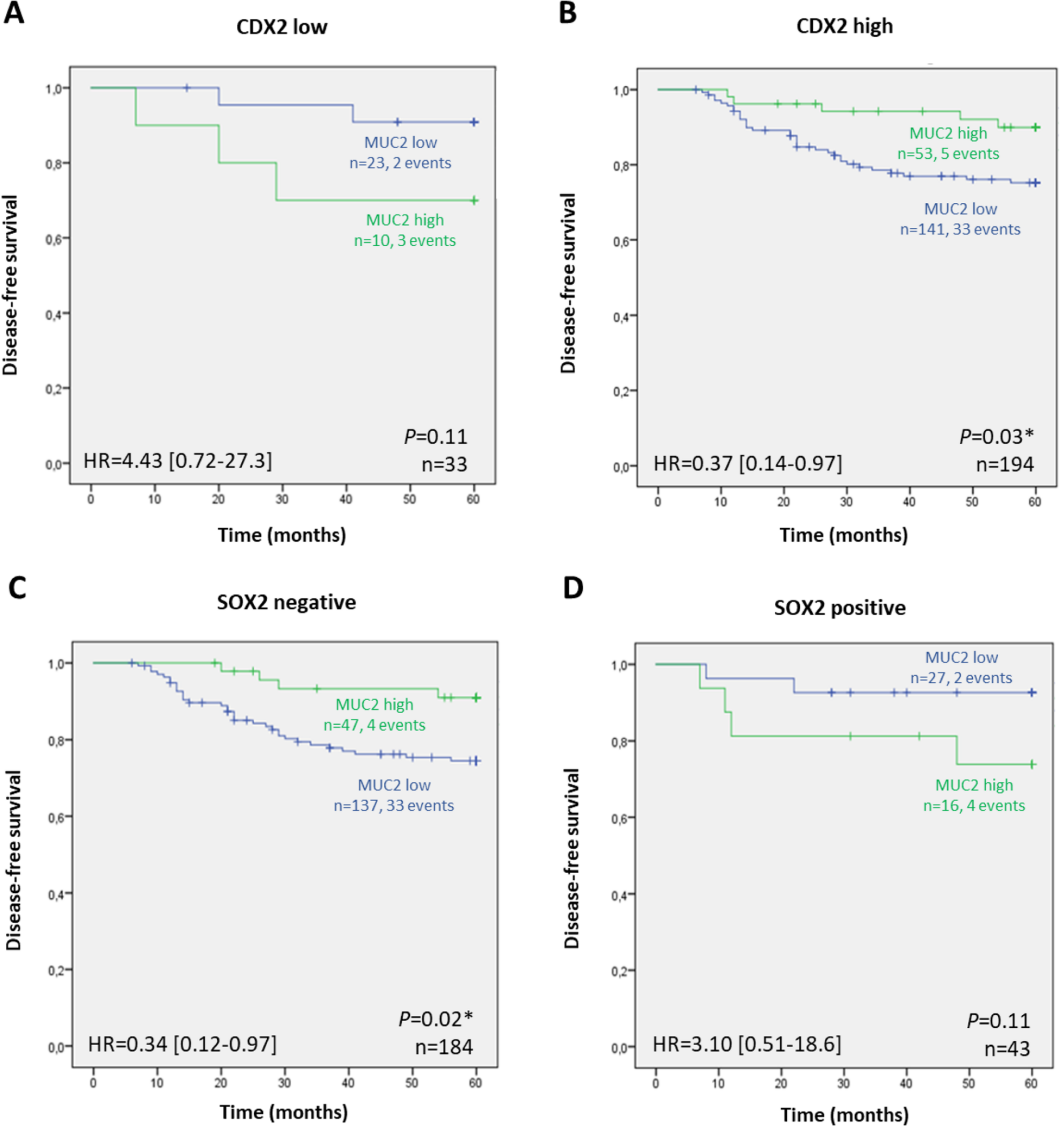
Kaplan-Meier curves showing the probability of disease-free survival, according to MUC2 and CDX2 expression (**a**, **b**) or MUC2 and SOX2 expression (**c**, **d**). The log-rank test was used to test for differences in survival between cases with high and low expression of MUC2, while univariable Cox regression (Wald) was used to generate hazard ratios (HR) and 95% confidence intervals (CI), after adjustment for sex and tumor grade

Then, we evaluated the prognostic relevance of the combined expression of CDX2, MUC2 and SOX2. A significant prognostic value was observed for MUC2 expression in cases that also expressed CDX2 (Fig. 3b; *P* = 0.03) and in cases without SOX2 expression (Fig. 3c; *P* = 0.02). In both situations, a higher DFS was observed in cases expressing MUC2. Combination of SOX2 and CDX2 expression did not reveal prognostic value (data not shown).

**Fig. 4:**
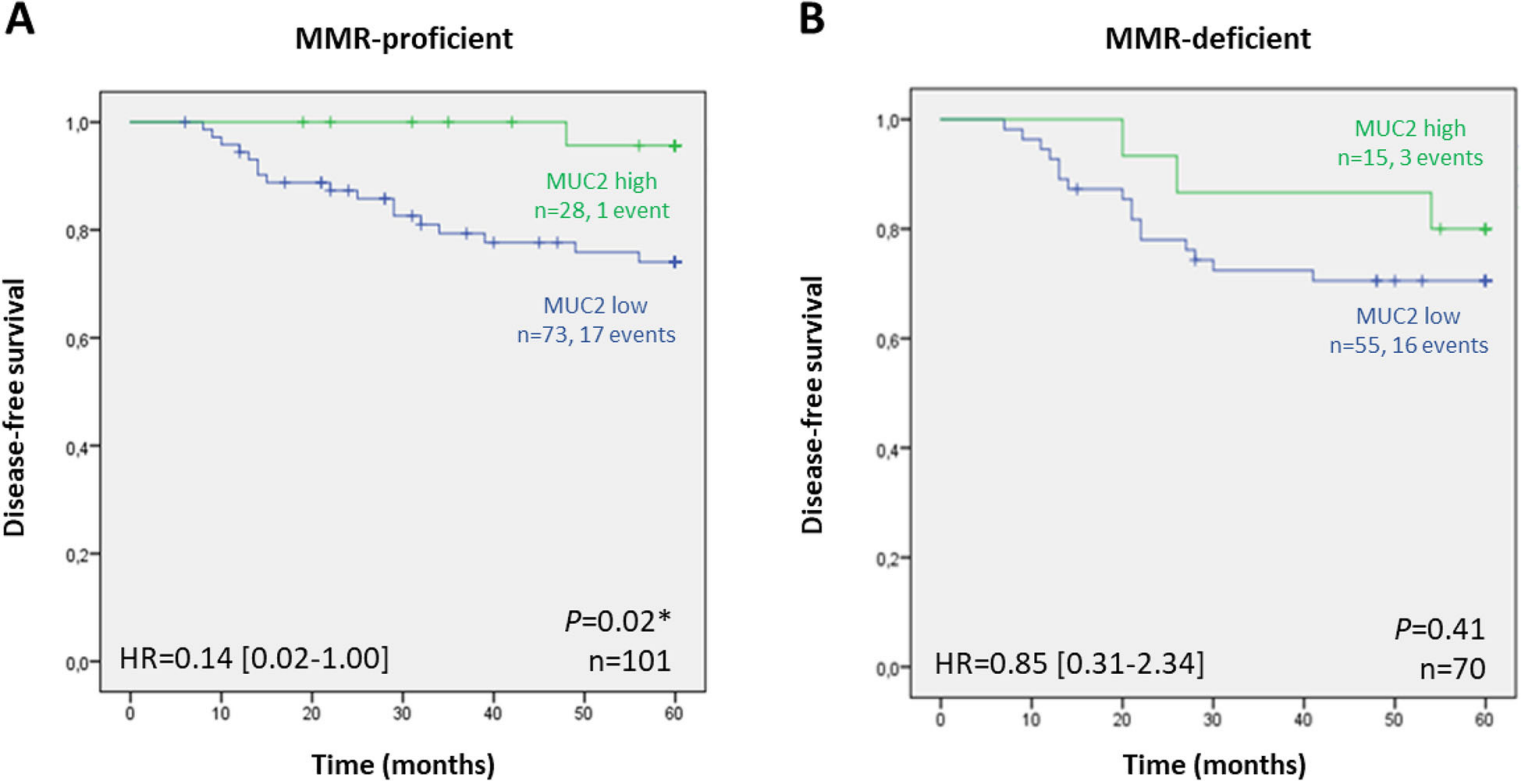
Kaplan-Meier curves showing the probability of disease-free survival, according to MUC2 expression and DNA MMR status: **a** MMR-proficient and **b** MMR-deficient cases. The log-rank test was used to test for differences in survival between cases with high and low expression of MUC2, while univariable Cox regression (Wald) was used to generate hazard ratios (HR) and 95% confidence intervals (CI), after adjustment for sex and tumor grade

Finally, a significantly worse prognosis was observed in cases with low MUC2 expression that were also MMR-proficient (Fig. 4a; *P* = 0.02).

### Predictive value of MUC2, CDX2 and SOX2 for response to adjuvant chemotherapy

**Fig. 5:**
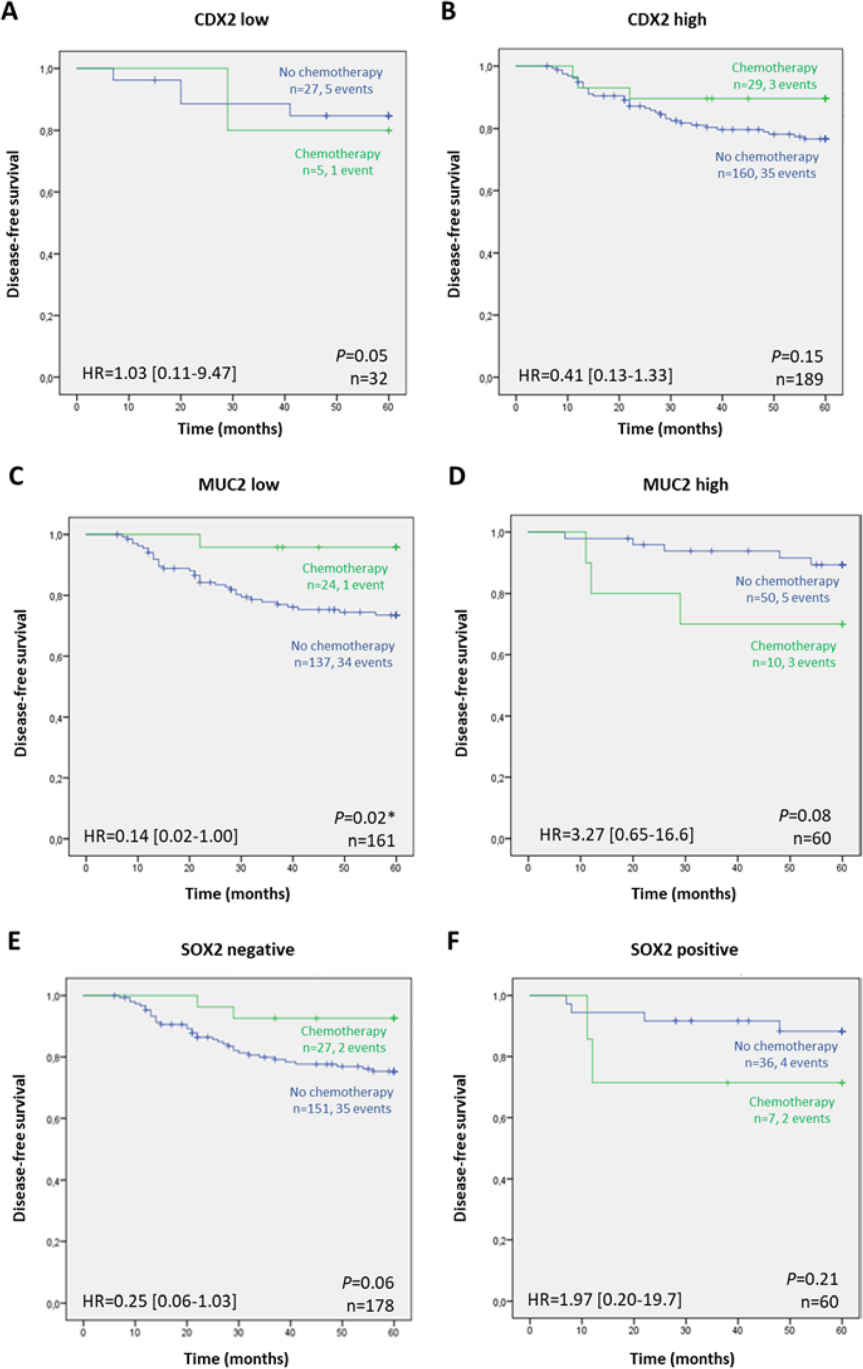
Kaplan-Meier curves showing the probability of disease-free according to treatment options in patients having tumors with: **a** low and **b** high expression of CDX2; **c** low and **d** high expression of MUC2; and **e** absence and **f** presence of SOX2, respectively. The log-rank test was used to test for differences in survival between treatment and absence of treatment with adjuvant chemotherapy, while univariable Cox regression (Wald) was used to generate hazard ratios (HR) and 95% confidence intervals (CI), after adjustment for sex and tumor grade

We then sought to study the value of CDX2, MUC2 and SOX2 as biomarkers of response to adjuvant chemotherapy. We observed a significantly better outcome in cases that had low levels of MUC2 when they were treated with chemotherapy, comparing with those not treated (Fig. 5c; *P* = 0.02). Patients with SOX2 negative tumors that were treated with adjuvant chemotherapy had a borderline significant lower probability of relapse than those not treated (Fig. 5e; *P* = 0.06). This difference was not observed in cases with SOX2 (Fig. 5f). Finally, CDX2 did not exhibit predictive value of response to chemotherapy, in this series of stage II CRC.

## Discussion

For patients diagnosed with stage II CRC, surgery has curative intention, yet 15–30% of these patients develop recurrent loco-regional disease or distant metastases within 5 years. Adjuvant chemotherapy, which is administered to a subset of the patients, does not significantly improve their survival [7, 16]. Thus, the current criteria used to select patients for adjuvant chemotherapy are clearly insufficient and this study was motivated by the need to identify biomarkers that, in a simple and consistent way, can be used to identify the patients that are at higher risk of relapse and could indeed benefit from chemotherapy. In our 227-patient series, we found that low expression of MUC2, in cases with high expression of CDX2, absence of SOX2 or MMR-proficiency, had a significantly worse prognosis. Moreover, cases with low expression of MUC2 showed a significantly clear benefit from treatment with adjuvant chemotherapy.

We found low levels of CDX2 in 14.5% of the stage II CRC patients and it was more often found in female patients, as already reported in Baba et al. (2009) [38] and Zhang et al. (2017) [39], in different stages of CRC, including in advanced cases. In our series, low expression of CDX2 also correlated with poorly and moderately differentiated tumors. This is in line with earlier reports that correlated loss of CDX2 expression with poorly differentiated tumors [15, 18, 40–42] and would be anticipated, since CDX2 is a major regulator of intestine-specific genes involved in cell differentiation [43, 44].

We also confirmed the positive association between CDX2 and the MMR status [41, 42]. In fact, CpG island methylator phenotype (CIMP)-positive CRC and cases with microsatellite instability (MSI) were reported to have methylation accompanied by decreased expression of CDX2 [15, 38, 41, 45]. However, whether loss of CDX2 expression plays a particularly active role in tumor progression in MSI/MMR-deficient tumors remains to be elucidated. It was reported that CDX2 loss does not confer a worse prognosis when considering MMR-deficient cases [46], although it predicted poor clinical outcome in stage II CRC cases with MSS phenotype [45]. According to previous studies that included stage II and stage III CRC cases [18, 24, 41, 42, 47], but contrarily to others [16, 45, 48, 49], we did not find prognostic nor predictive value for CDX2 expression in stage II CRC. Yet, this could be, at least in part, due to the size of our series with consequent low number of tumors with loss of CDX2 expression, low number of recurrence events as well as low number of patients treated with chemotherapy. Other limitations of our study include the use of only one hospital series and shortcomings related with the use of subjective scoring systems and TMAs for immunohistochemical protein analyses. This adds up to the relevance of the results obtained with MUC2, suggesting it could be a better marker in early CRC stages. MUC2 mucin is abundantly expressed by goblet cells in normal CRC mucosa yet it was negative or lowly expressed already in 72.2% of the stage II CRC tumors. Furthermore, although not statistically significant, there was a clear tendency for a lower DFS when tumors have low MUC2 expression, in accordance with the results shown in a very recent paper from Cecchini et al. (2019) [24], in which the authors studied 210 cases of stage II CRC and showed that absence of MUC2 expression was associated with reduced survival. Concordantly, Kang et al. (2011) [20] and Betge et al. (2016) [23] studied 229 and 381 cases with CRC in stages II and III and observed a significantly decreased OS in cases where MUC2 expression was lost. In particular, Ohlsson et al. (2012) [21] studied MUC2 mRNA levels in mesenteric lymph nodes of CRC and concluded that patients with a low MUC2/CEA ratio had a significantly smaller average survival. Loss of MUC2 expression might be a surrogate marker of loss of intestinal differentiation and might occur through mechanisms that do not involve regulation by transcription factors. When we combined the information regarding the expression of CDX2 or SOX2 transcription factors with the expression of MUC2, we observed a significantly lower DFS for cases exhibiting low levels of MUC2 and high levels of CDX2 or absence of SOX2. In addition, a significantly worse prognosis was observed for cases with low MUC2 expression that were also MMR-proficient, corroborating the observation of Betge et al. (2016) [23]. Lugli et al. (2007) [50] otherwise showed, in a large series of 1420 patients, that loss of MUC2 was associated with poorer survival in both MMR-proficient and MLH1-deficient tumors from all stages. It is relevant to note that the percentage of patients with MMR-deficient tumors in our series (43.5%) was higher than usual (around 15–20%) [51, 52]. In this study, MMR was assessed by determining the expression of the MMR genes using IHC in tissue microarrays (TMAs). This fact might increase the number of negative cases for the expression of MMR genes, since the area of the tumor analysed in TMAs is smaller. However, two studies performed in stage II-III colon cancer, that analyzed the expression of 4 MMR genes by IHC, but not using TMAs, also describe high levels of MMR-deficiency, respectively 33 and 30% [53, 54]. Other explanations for the high percentage of MMR-deficient patients may be linked to older age, large proportion of poorly differentiated tumors, location in the proximal colon and lympho-vascular invasion. We cannot exclude that this series has a relatively higher percentage of patients with Lynch syndrome [55] however this is speculative.

In addition, even with a relatively small and rather homogeneous series, we could identify the predictive significance of MUC2 expression in the response to adjuvant chemotherapy. The results obtained suggested that patients with low levels of MUC2 expression in the tumor respond better when they are treated, meaning that they can benefit more from adjuvant treatment than cases with high expression of MUC2, where no significant difference was observed between patients treated and those not treated with adjuvant therapy. This could be related with the general finding that low MUC2 expression is associated with worse prognosis, thus the treatment of these tumors needs to be more aggressive to be effective.

On the other end, there is mucinous differentiation, which is characterized by the abundant expression of MUC2 and other 11p15.5 mucins, associated with both CpG island methylator phenotype and microsatellite instability in CRC. Walsh et al. (2013) [56] reported an association between expression of MUC2 (and also MUC5AC, MUC5B, and MUC6) and the presence of somatic *BRAF*^*V600E*^ mutation, CIMP, MSI, MMR-deficiency and loss of CDX2 expression in a large series of CRC. Several studies report that mucinous adenocarcinoma is more likely associated with advanced stages in CRC and less responsive to chemotherapy, comparing to non-mucinous adenocarcinoma [57, 58]. The prognostic value of the mucinous histological subtype remains controversial, and some studies, including a very recently published large population-based study, disclosed that there was no significant difference in survival between these two entities in stage II CRC [59].

SOX2 is not expressed in the normal intestinal epithelium and it has been reported that it is amplified in digestive cancers [32, 33]. We found de novo expression of SOX2 in 18.9% of the stage II CRC patients. In our study, SOX2 expression was not correlated with poor differentiation nor with *BRAF*^*V600E*^ mutation, contrarily to what has been described by Lundberg et al. (2014) [31] in a series of 441 CRC patients encompassing all stages. SOX2 is anticipated to have a vital role in CRC, since it is broadly related with stemness, growth, invasion and metastasis [31, 60–62]. SOX2 overexpression has indeed been co-related with tumor progression, disease recurrence and poor OS [31, 32, 63]. However, in our stage II CRC cohort, SOX2 expression did not show any prognostic value by itself, which can indicate stage-dependency, consistent with observations in previous studies [31]. However, stage II CRC patients with expression of the stem-like markers CD44, LGR5, SOX2 and OCT4 in the tumors had a significantly worse prognosis compared to those with lower expression and showed a tendency to benefit from adjuvant treatment [64]. Takeda et al. (2018) have shown in vitro that SOX2-positive cells presented chemoresistance to oxaliplatin and 5-FU, demonstrating higher expression of cancer stem cell markers besides typical asymmetric cell division [65]. In our study, a borderline significant trend to a higher benefit from chemotherapy (*P* = 0.06) was instead observed for the SOX2 negative cases.

## Conclusions

In our cohort of stage II colorectal cancer patients, we identified a role for MUC2 as predictor of response to adjuvant chemotherapy. This observation supports that MUC2 is involved in resistance to fluorouracil-based adjuvant chemotherapy and might be a promising predictive biomarker in stage II CRC patients.

## Supplementary Information

**Additional file 1: Table S1.** The REMARK checklist.

**Additional file 2: Table S2**. Disease-free survival univariable Cox regression analysis in our stage II CRC.

## Data Availability

The datasets used and/or analysed during the current study are available from the corresponding author on reasonable request.

## Abbreviations

5-FU: Fluorouracil
CDX2: Caudal type homeobox 2 transcription factor
CHSJ: Centro Hospitalar S. João
CI: Confidence intervals
CIMP: CpG island methylator phenotype
CRC: Colorectal cancer
DFS: Disease-free survival
HE: Haematoxylin and eosin
HR: Hazard ratios
IHC: Immunohistochemistry
MLH1: MutL homolog 1, mismatch repair gene
MMR: Mismatch repair
MSH2: MutS homolog 2, mismatch repair gene
MSH6: MutS homolog 6, mismatch repair gene
MSI: Microsatellite instability
MSS: Microsatellite stable
MUC: Mucin
MUC2: Mucin 2, oligomeric mucus/gel-forming
OS: Overall survival
PMS2: PMS1 homolog 2, mismatch repair gene
SOX2: Sex-determining region Y (SRY)-box transcription factor 2
TFs: Transcription factors
TMAs: Tissue microarrays
χ^2^: Chi-square
