# Supplementary material for: Prognostic significance of MUC2, CDX2 and SOX2 in stage II colorectal cancer patients
Source: BMC Cancer. 2021 Apr 6;21:359. doi: 10.1186/s12885-021-08070-6 (PMC8025574; doi:10.1186/s12885-021-08070-6)
Supplement: Supplementary file 2 — Additional file 2: Table S2. Disease-free survival univariable Cox regression analysis in our stage II CRC. [file 12885_2021_8070_MOESM2_ESM.docx]

**Table S2 - Disease-free survival univariable Cox regression analysis in our stage II CRC.**

|  | **Number of events** | **Univariable analysis** | | |
| --- | --- | --- | --- | --- |
|  |  | **HR** | **95% CI** | ***P*** |
| **Histopathological grade (n=224)** |  |  |  |  |
| G1 | 4 | 1 |  |  |
| G2 | 210 | 0.681 | 0.094-4.949 | 0.704 |
| G3 | 10 | 0.371 | 0.023-5.930 | 0.483 |
| **Tumor Location (n=224)** |  |  |  |  |
| Proximal colon | 75 | 1 |  |  |
| Distal colon | 107 | 1.282 | 0.653-2.518 | 0.470 |
| Rectum | 42 | 0.803 | 0.305-2.113 | 0.657 |
| **Mismatch repair status (n=209)** |  |  |  |  |
| MMR-proficient | 118 | 1 |  |  |
| MMR-deficient | 91 | 1.510 | 0.817-2.790 | 0.188 |
| ***BRAF*^V600E^**  **(n=216)** |  |  |  |  |
| WT | 186 | 1 |  |  |
| MUT | 30 | 0.838 | 0.329-2.131 | 0.710 |
| **CDX2** |  |  |  |  |
| low | 33 | 1 |  |  |
| high | 194 | 1.371 | 0.539-3.483 | 0.508 |
| **MUC2** |  |  |  |  |
| low | 164 | 1 |  |  |
| high | 63 | 0.551 | 0.255-1.187 | 0.128 |
| **SOX2** |  |  |  |  |
| - | 184 | 1 |  |  |
| + | 43 | 0.684 | 0.289-1.620 | 0.388 |
